# Effects of minimum legal drinking age on alcohol and marijuana use: evidence from toxicological testing data for fatally injured drivers aged 16 to 25 years

**DOI:** 10.1186/s40621-014-0032-1

**Published:** 2015-01-12

**Authors:** Katherine M Keyes, Joanne E Brady, Guohua Li

**Affiliations:** 1 Department of Epidemiology, Columbia University, Mailman School of Public Health, 722 West 168th Street, Suite 503, New York, NY 10032 USA; 2 Department of Anesthesiology, Columbia University, New York, NY USA

**Keywords:** Marijuana, Alcohol, Substitution, Complements, Minimum legal drinking age, FARS

## Abstract

**Background:**

Alcohol and marijuana are among the most commonly used drugs by adolescents and young adults. The question of whether these two drugs are substitutes or complements has important implications for public policy and prevention strategies, especially as laws regarding the use of marijuana are rapidly changing.

**Methods:**

Data were drawn from fatally injured drivers aged 16 to 25 who died within 1 h of the crash in nine states with high rates of toxicology testing based from 1999 to 2011 on the Fatality Analysis Reporting System (N = 7,191). Drug tests were performed using chromatography and radioimmunoassay techniques based on blood and/or urine specimens. Relative risk regression and Joinpoint permutation analysis were used.

**Results:**

Overall, 50.5% of the drivers studied tested positive for alcohol or marijuana. Univariable relative risk modeling revealed that reaching the minimum legal drinking age was associated with a 14% increased risk of alcohol use (RR = 1.14, 95% CI: 1.02 to 1.28), a 24% decreased risk of marijuana use (RR = 0.76, 95% CI: 0.53 to 1.10), and a 22% increased risk of alcohol plus marijuana use (RR=1.22, 95% CI: 0.90 to 1.66). Joinpoint permutation analysis indicated that the prevalence of alcohol use by age is best described by two slopes, with a change at age 21. There was limited evidence for a change at age 21 for marijuana use.

**Conclusions:**

These results suggest that among adolescents and young adults, increases in alcohol availability after reaching the MLDA have marginal effect on marijuana use.

## Background

Alcohol and marijuana are among the most commonly used drugs by adolescents and young adults in the United States (US) (Substance Abuse and Mental Health Services Administration, 2011). Use is associated with substantial morbidity and mortality for young people, especially motor vehicle crash fatality, which is a leading cause of death among those 18 to 25 in the US (Heron, 2013). In 2012, more than 33,500 individuals died in motor vehicle crashes (NHTSA, 2013), and based on the most recent data available, about 14% of drivers involved in fatal crashes are under the influence of alcohol, drugs, or medication at the time of a fatal crash (FARS, 2011). Current estimates indicate that, in states that routinely test drivers who die within 1 h of a crash, more than half of these drivers are under the influence of alcohol and/or other drugs at the time of death (Brady and Li, 2013). Understanding and preventing injuries from motor vehicle crashes, especially among young adults, remains an important public health priority. Policy change has proven efficacious in reducing the harm of alcohol-impaired driving (Cohen and Einav, 2003; Shults et al. 2001; Task Force on Community Preventive, 2001; Task Force on Community Preventive Services, 2001, 2005), including raising the minimum legal drinking age (MLDA) (Plunk et al. 2013; Subbaraman and Kerr, 2013; Wagenaar and Toomey, 2002), lowering the legal blood alcohol content (BAC) limits for drivers (Mercer et al. 2010; Wagenaar et al. 2007), and increasing the tax and price of alcoholic beverages (Wagenaar et al. 2010).

Policies related to alcohol, as well as other substances, both in the US and more broadly, remain an open area of debate and controversy. Most recently, the Amethyst Initiative (Amethyst Initiative, 2008 ), a coalition of more than 130 university presidents in the US, advocated for a reduction in the minimum legal drinking age (MLDA) to age 18, with advocates suggesting that such a policy change could reduce harms associated with illegal drug use among young adults by giving them legal access to alcohol. The evidence for such a claim, however, remains unclear (DeJong and Blanchette, 2014; Fitzpatrick et al. 2012; Nelson et al. 2010). Other policies related to alcohol such as changes in the minimum acceptable BAC to operate a vehicle, changes in tax, import, and export policy remain in flux both in the US and worldwide (Rehm and Greenfield, 2008). More broadly, policies related to other substances of potential abuse are changing. For example, marijuana policy is undergoing tremendous shifts toward increased access, with 23 states now approving of medical use in some form and two states (California and Washington) approving legislation to legalize marijuana for adult recreational use (Hoffmann and Weber, 2010).

When one substance becomes more legally accessible, what happens to the prevalence of other substances? This question is often framed in economics in terms of whether two goods are complements or substitutes. A complement good is one in which demand increases as a function of the availability of a related good; in contrast, a substitute good is one in which use decreases with increased availability of a related good (Nicholson, 1998). The issue of whether alcohol and marijuana are complements or substitutes has important direct policy and public health implications. If marijuana and alcohol are complement goods, we can expect increased marijuana use and perhaps other drugs of abuse with increased access to alcohol. This would portend increases in intentional and unintentional injury, increased rates of dependence, and other potential consequences. On the other hand, if alcohol and marijuana are substitutes, increased alcohol availability may have an unintended *benefit* of reduced harm associated with marijuana use (though potential harms associated with alcohol use may balance any potential benefit).

Substantial economic literature has examined whether alcohol and marijuana operate as complement or substitute goods after a policy and/or price change. When prices (Cameron and Williams, 2001; Chaloupka and Laixuthai, 1997; Farrelly et al. 2001; Pacula, 1998; Saffer and Chaloupka, 1999; Williams et al. 2004), taxes (Pacula, 1998), policies/laws (Anderson et al. 2013; Cameron and Williams, 2001; Chaloupka and Laixuthai, 1997; DiNardo and Lemieux, 2001; Farrelly et al. 2001; Pacula, 1998; Saffer and Chaloupka, 1999; Thies and Register, 1993; Williams et al. 2004), and college campus alcohol polices (Williams et al. 2004) have been examined, the evidence to date has not pointed to a clear substitution or complementary relation, even within the study (e.g., (Chaloupka and Laixuthai, 1997; Pacula, 1998; Pacula et al. 2013; Thies and Register, 1993)). Inference has been limited, however, by data quality (e.g., drug and alcohol price information is subject to substantial error) and unobserved confounding (e.g., states that decriminalize marijuana may have generally less negative attitudes toward substance use). Further, self-reported alcohol and marijuana use is also subject to reporting error (Buchan et al. 2002; Del Boca and Darkes, 2003); no studies to date, to our knowledge, have used toxicological data on alcohol and marijuana positivity to assess drug use in studies assessing whether these substances are economic substitutes or complements.

In contrast to price and taxes, minimum legal drinking age (MLDA) laws have well-documented effects on alcohol consumption and alcohol-associated injury (McCartt et al. 2010; Plunk et al. 2013; Subbaraman and Kerr, 2013; Wagenaar and Toomey, 2002), especially among young adults. As such, examining whether marijuana use increases or decreases as individuals age into legal drinking is an opportunity to test whether marijuana is a substitute or complement good for alcohol. Four studies have examined the effects of MLDA on demand for alcohol and marijuana, with conflicting results (Crost and Guerrero, 2012; Crost and Rees, 2013; DiNardo and Lemieux, 2001; Thies and Register, 1993; Yoruk and Yoruk, 2011). Perhaps the most rigorous examinations in recent literature have utilized a regression discontinuity (RD) design (Shadish et al. 2002). The RD design is a quasi-experimental approach that exploits the observation that birth dates are relatively randomly distributed, thus individuals right below the MLDA and individuals right above the MLDA are similar to each other on many risk factors for alcohol use except legal drinking status. The slope of the regression line between age and marijuana when (a) alcohol is legally accessible (age 21 and beyond) to the slope of the regression line when (b) alcohol is not legally accessible (prior to age 21) is then compared for evidence of discontinuity (i.e. non-linearity) in the slope of the line. Thus, the counterfactual question raised is, holding all else equal, would the rate of marijuana use continue to increase, or instead decrease, when alcohol becomes available.

Existing studies on the relation between age and alcohol use indicates that there is a positive and relatively linear upward trend in use from the late teens through the mid-20s (Chen and Jacobson, 2012; Jager et al. 2013). The relation between age and marijuana use is less linear, with an upward trend in the late teens and then a flattening of the slope in the early 20s (Chen and Jacobson, 2012; Jager et al. 2013). While these trajectories differ in the magnitude of the slope at the population level, at the individual level, alcohol and marijuana use are substantially correlated (Kandel et al. 1992). That is, those who use alcohol are approximately 2 to 3 times more likely to marijuana. As such, examination of joint trajectories of alcohol and marijuana use through the developmental young adult period when alcohol becomes legal is critical. Given that alcohol use is expected to increase after age 21, if alcohol and marijuana are economic complements, we would also expect that marijuana use would *increase* among alcohol users but that marijuana use in the absence of alcohol use would *decrease*. Conversely, if alcohol and marijuana are economic substitutes, we would expect that marijuana use would *decrease* among alcohol users and that marijuana use in the absence of alcohol use would *increase*. To date, existing studies utilizing MLDA as an instrument in the regression discontinuity design have not considered these joint effects, leaving open questions remaining about the effects of MLDA on marijuana use. Further, these existing studies have had conflicting results. Using aggregated state-level national US data, Crost and Guerrero (2012) found that individuals over 21 have higher self-reported past-month days of alcohol use and lower past-month days marijuana use, consistent with a substitution effect. Using nationally representative longitudinal data, Yoruk and Yoruk (2011) and Crost and Rees (2013) also document a decrease in marijuana use after age 21. However, whether this effect is robust in samples with high-risk of alcohol and marijuana, such as fatally injured drivers, remains unknown. Examination of these effects in high-risk samples and based on toxicological testing data is critical, as these samples represent the groups with the most adverse health consequences of substance use and are less susceptible to information bias. If marijuana use decreases after age 21 mostly in subgroups of the population with low risk of heavy use or health consequences, but increases among those at high risk, the public health strategy to reduce harms associated with substance use during the transition to adulthood will need to be modified.

In summary, existing literature on how changes in alcohol availability affects marijuana use remains indeterminate, and causal inference approaches such as regression discontinuity designs have the potential to inform this literature. However, no such studies have used toxicological information on alcohol and marijuana positivity in high-risk groups such as crash decedents, and no studies have examined joint trajectories of alcohol and marijuana use. Using the data for drivers aged 16 to 25 years who were fatally injured within 1 h of the crash in 13 states where toxicological testing was performed on a routine basis during 1999 to 2010 (n = 7,191), we assessed the effects of MLDA (i.e., 21 years) on: (1) alcohol plus marijuana use; (2) alcohol use only; and (3) marijuana use only using a regression discontinuity design.

## Methods

### Data source

Data were drawn from the Fatality Analysis Reporting System (FARS), a census of fatal traffic crashes occurring within the United States maintained by the National Highway Traffic Safety Administration (Hargutt et al. 2011). All crashes involving a motor vehicle traveling on a public road and resulting in a fatality within 30 days are included in the census. Detailed data from police reports, state administrative files, and medical records are collected on circumstances, vehicles, and people involved in the crash. Trained analysts using standard forms and protocols maintain the records and specified quality control procedures are rigorously implemented (National Highway Traffic Safety Administration, 2010).

Drivers between the age of 16 and 25 at the time of death were included. We include data from states that performed toxicological testing on more than 85% of their fatally injured drivers who died within 1 h of the crash (California, Connecticut, Hawaii, Illinois, New Hampshire, New Jersey, Rhode Island, Washington, and West Virginia) from 1999 to 2011. We included only drivers who died within 1 h of the crash because the validity of drug and alcohol testing data may be compromised. Alcohol and drugs taken before the crash might be undetectable if tested more than 1 h after the crash, rendering false negatives. Further, drugs administered after the crash by medical personnel may be detected, rendering false positives. Despite higher than 85% testing rates, data from New Mexico were excluded from the study sample because test results recorded in FARS for this state were deemed unreliable due to the low number of drivers positive for drugs (NHTSA, 2010). Drivers testing positive for drugs other than alcohol and/or marijuana were excluded (n = 1,525). Of the remaining 7,905 drivers fatally injured between 1999 and 2011, 714 (9.0%) were excluded from the analysis due to the lack of drug testing data. Drivers who survived more than 1 h after the crash (n = 3,981) or with missing time of death information (n = 197) were excluded from this study because of concerns about the accuracy and reliability of drug testing data for these drivers.

### Measures

#### Driver characteristics

Data are routinely collected on demographics of the fatally injured driver including age (in years), sex, race, and ethnicity. Race/ethnicity was missing on 9.6% of the injuries and was categorized into White (86.4%) versus non-White.

### Crash characteristics

We included two characteristics of the crash itself in the analysis: the number of occupants of the vehicle and the number of fatalities, as they are associated with age of the fatally injured driver (Tefft et al. 2012), thus potentially important characteristics to assess within the context of the effects of MLDA. Number of occupants was categorized into 1 (64.8%), 2 (21.7%), and 3 or more (13.5%). Data on number of vehicle occupants were missing on 11.9% of the sample. Of those with data, number of deaths was categorized into 1 (86.3%) and more than 1 (13.7%). We also controlled for a categorical indicator of the state where the crash occurred: California (N = 4,777, 54.7%), Connecticut (N = 402, 4.6%), Hawaii (N = 95, 1.1%), Illinois (1,211, 13.9%), New Hampshire (N = 168, 1.9%), New Jersey (N = 583, 6.7%), Rhode Island (N = 120, 1.4%), Washington (N = 867, 9.9%), West Virginia (N = 516, 5.9%), as well as year of the crash. Further, we separately controlled for whether the state had a medical marijuana law, as some data indicates that marijuana use is higher in states with medical marijuana laws (Cerda et al. 2012; Wall et al. 2011). California and Washington had some form of MML for the entirety of the study period; Connecticut, Illinois, New Hampshire, and West Virginia did not have an MML for the entirety of the study period. For remaining states, decedents were coded as in a state with an MML based on when the law was passed: Hawaii (2000), New Jersey (2010), and Rhode Island (2006).

### Drug and alcohol test results

Drug tests were performed using chromatography and radioimmunoassay techniques based on blood and/or urine specimens (Centers for Disease, C., Prevention 2006; Li et al. 2011). Drugs were categorized according to the FARS coding manual (National Highway Traffic Safety Administration, 2008) and grouped into the following categories: alcohol and cannabinoid, alcohol only, cannabinoid only, and neither.

Drug testing protocols might vary from state to state (The Walsh Group, 2002; Walsh et al. 2004). The testing methods and specimens might not be exactly the same across the states. The possible bias resulting from different specimens, however, was unlikely to pose a serious threat to the validity of this study given that 94% of the study sample had at least one test based on a blood specimen. However, we note that we controlled for state in adjusted models to ensure that results were not biased by state variation in protocols.

### Statistical analysis

First, we estimated the prevalence of alcohol and marijuana involvement by single year of age among fatality injured drivers and estimated the percentage change in alcohol and marijuana involvement at each age increase. BAC ≥ 0.01 g/DL was considered alcohol positive.

Second, we examined whether the percentage change by year substantially changed the slope of the relation between age and alcohol/marijuana use using the National Cancer Institute’s Joinpoint software (Kim et al. 2000). We estimated ‘points of inflection’, that is, specific ages in which the slope of the association between age and drug use significantly changes. The Joinpoint software estimates a series of permutations with increasing number of inflection points and indicates the minimum number necessary such that additional inflection points do not improve model fit.

Finally, we estimated three relative risk regression models using three different outcomes: (1) alcohol use plus marijuana use versus no use; (2) alcohol use only versus no use; and (3) marijuana use only versus no use. Regression models for discontinuity regression included centered age, post-21 indicator, and their interaction:

1 $$ \mathrm{Log}\left(Y=1\Big|X\right)= \log \mu ={\beta}_0+{\beta}_1{T}_i+f\left({\mathrm{age}}_i\right)+{\beta}_2\left({T}_i*{\mathrm{age}}_i\ \right) $$

where *Y*_*i*_is each of our three outcomes, *T*_*i*_ is the post-21 indicator (legal drinker, yes/no), and *f*(age_*i*_) is a centered age function (calculated as the difference in respondent’s age from age 21 in number of years). From this equation, we estimated the risk ratio for the effect of turning 21 (aging into legal drinking) on alcohol use, marijuana use, and alcohol plus marijuana use.

We then explored the effect of control covariates on the association between MLDA and alcohol/marijuana use including driver sex, race/ethnicity, number of occupants in the vehicle, number of deaths in the incident, year, state, and whether the state had an MML. There were 854 missing values for vehicle occupancy (11.9%), 716 missing values for race (9.6%), and 1,343 missing on either occupancy or race (18.6%), handled in the analysis with list-wise deletion controlling for these covariates separately.

## Results

**Figure 1:**
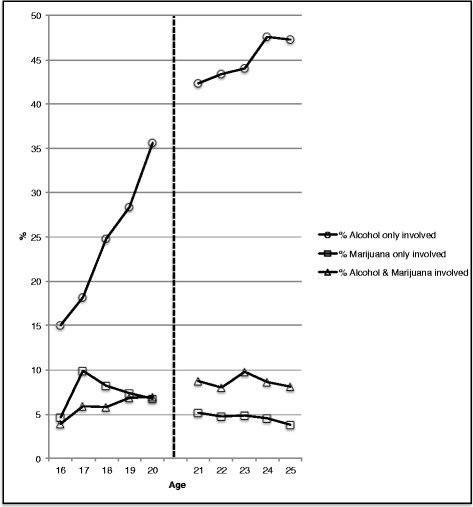
**Prevalence of alcohol and marijuana positivity in drivers.** Prevalence of alcohol and marijuana positivity in drivers who died within 1 h of crash by age, FARS, selected states, 1999 to 2010.

As shown in Table 1, 50.3% of the drivers studied tested positive for alcohol or marijuana (36.8% for alcohol only, 5.9% for marijuana only, and 7.6% for both drugs). Data on single drug use indicated that the prevalence of alcohol use only increased monotonically from 15.0% at age 16 to 35.6% at age 20 years, and continued to rise at a slower pace after age 20. The prevalence of marijuana increased slightly from 4.6% at age 16 to 6.7% at age 20 and monotonically decreased after age 20. The prevalence of combined use of alcohol and marijuana increased progressively from age 16 to 20 before leveling off. Examining the percentage change from year to year, for alcohol without marijuana use, the percentage change was positive in every year, with the highest change between age 19 and 20 (increase of 7.2%), and another large change from 20 to 21 (increase of 6.7%). For marijuana without alcohol use, the percentage change was mostly negative, with the largest negative decrease between 17 and 18 (decrease of 1.7%) and another large change from 20 to 21 (decrease of 1.5%). For alcohol and marijuana use, percentage changes were mostly positive, with large changes occurring between age 16 and 17 (increase of 2%), 20 and 21 (increase of 1.8%), and 22 and 23 (increase of 1.8%). In Figure 1, we graph the prevalence of alcohol plus marijuana use, alcohol only, and marijuana only positivity among deceased drivers, with a cut point at age 21 to visually display the potential for discontinuity across the timespan of the study.

**Table 1:** **Prevalence of alcohol and marijuana in drivers who died within 1 hour of crash by age, FARS, selected states, 1999 to 2010**

**Age**	**Number of drivers tested**	**Percent alcohol only involved**	**Absolute change in percent for each year of age**	**Percent Marijuana only involved**	**Absolute change in percent for each year of age**	**Alcohol and marijuana involved**	**Absolute change in percent for each year of age**
16	280	15		4.6		3.9	
17	473	18.2	3.2	9.9	5.3	5.9	2
18	770	24.8	6.6	8.2	−1.7	5.8	−0.1
19	825	28.4	3.6	7.4	−0.8	6.8	1
20	811	35.6	7.2	6.7	−0.7	6.9	0.1
21	973	42.3	6.7	5.2	−1.5	8.7	1.8
22	875	43.4	1.1	4.7	−0.7	8	−0.7
23	769	44	0.6	4.8	0.1	9.8	1.8
24	758	47.6	3.6	4.5	−0.3	8.6	−1.2
25	657	47.3	−0.3	3.8	−0.7	8.1	−0.5
Total	*7,191*	36.8		5.9		7.6

We then used Joinpoint regression analysis to examine whether there is evidence for discontinuity in the relation between age and alcohol/marijuana use. Among those who consumed alcohol alone (without marijuana), Joinpoint analysis indicated that the best model fit was two slopes (comparing a two slope model to a one slope model, the p-value was <0.001 in favor of the two slope model), with an inflection point at age 21 (95% confidence interval, age 19 to age 22). Before age 21, the relation between age and alcohol use is significant and positive (B = 0.21, SE = 0.01, *p* < 0.001). After age 21, the relation is significant and negative (B = −0.18, SE = 0.02, *p* < 0.001). For combined alcohol and marijuana, there was marginal support for two slopes (comparing a two slope model to a one slope model, the *p*-value was 0.053 in favor of the two slope model); however, the inflection point was at age 23 (95% confidence interval for the inflection point age 18 to 25). Before age 23, the relation between age and concurrent alcohol and marijuana use is significant and positive (B = 0.11, SE = 0.01, *p* = 0.004). After age 23, the relation is null (B = −0.21, SE = 0.17, *p* = 0.26). For marijuana, a one slope model best fits the data (comparing a two slope model to a one slope model, the *p*-value was 0.09 suggesting that the two slope model did not substantially improve model fit). The relation between age and marijuana positivity exhibited a negative slope (B = −0.08, SE = 0.03, *p* = 0.03).

In Table 2, we test whether the prevalence of alcohol use only, marijuana use only, and alcohol plus marijuana use changed before and after age 21 using regression models. That is, we tested the magnitude of the discontinuity in prevalence at age 21. Univariable relative risk modeling revealed that reaching the minimum legal drinking age was associated with a 14% increased risk of alcohol use (RR = 1.14, 95% CI: 1.02 to 1.28), a 24% decreased risk of marijuana use (RR = 0.76, 95% CI: 0.90 to 1.66), and a 22% increased risk of alcohol plus marijuana use (95% CI: 0.90 to 1.66). In Table 3, we test the robustness of these effects, controlling for covariates separately and together. Controlling for each covariate separately did not change the results. When all covariates were controlled simultaneously, the magnitude of the results did not change (e.g., RR = 1.12 for alcohol only, RR = 0.80 for marijuana only, and RR = 1.24 for alcohol plus marijuana), though confidence intervals were wider due to approximately 19% missing data.

**Table 2:** **Estimated relative risks of alcohol and marijuana use in fatally injured drivers 1999 to 2010 associated with MLDA**

**Drug**	**RR**	**95% confidence interval**
Alcohol only	1.14	1.02 to 1.28
Marijuana only	0.76	0.53 to 1.10
Alcohol and marijuana	1.22	0.90 to 1.66

**Table 3:** **Estimated relative risks of alcohol and marijuana use in fatally injured drivers 1999–2010 associated with MLDA, adjusting for covariates**

**Covariate**	**Drug**	**RR**	**95% confidence interval**
Vehicle occupants (N = 6,337^a^)	Alcohol only	1.13	1.01 to 1.26
	Marijuana only	0.78	0.53 to 1.16
	Alcohol and marijuana	1.21	0.87 to 1.70
Sex (N = 7,191)	Alcohol only	1.13	1.01 to 1.26
	Marijuana only	0.75	0.53 to 1.08
	Alcohol and marijuana	1.22	0.90 to 1.65
Number of deaths (N = 7,191)	Alcohol only	1.14	1.02 to 1.28
	Marijuana only	0.76	0.53 to 1.10
	Alcohol and marijuana	1.22	0.90 to 1.66
Race (N = 6,475^b^)	Alcohol only	1.14	1.01 to 1.28
	Marijuana only	0.77	0.52 to 1.13
	Alcohol and marijuana	1.30	0.95 to 1.80
Year (N = 7,191)	Alcohol only	1.14	1.02 to 1.28
	Marijuana only	0.75	0.52 to 1.07
	Alcohol and marijuana	1.21	0.90 to 1.64
State (N = 7,191)	Alcohol only	1.14	1.02 to 1.27
	Marijuana only	0.75	0.53 to 1.08
	Alcohol and marijuana	1.19	0.88 to 1.61
Hispanic ethnicity (N = 6,475^b^)	Alcohol only	1.14	1.01 to 1.28
	Marijuana only	0.78	0.53 to 1.14
	Alcohol and marijuana	1.30	0.95 to 1.79
State allows medical use of marijuana (N = 7,191)	Alcohol only	1.14	1.02 to 1.28
	Marijuana only	0.77	0.54 to 1.10
	Alcohol and marijuana	1.22	0.90 to 1.65
All covariates (N = 5,848^c^)	Alcohol only	1.12	0.99 to 1.26
	Marijuana only	0.80	0.53 to 1.21
	Alcohol and marijuana	1.24	0.87 to 1.75

^a^854 missing data points.

^b^716 missing data points.

^c^Listwise deletion for missing data on vehicle occupants and race rendered 1,343 missing data points.

## Discussion

The present study documents that approximately 50% of fatally injured drivers aged 16 to 25 years tested positive for alcohol, marijuana, or both. We tested whether there was evidence for substitution or complement use at the discontinuity of age 21, when drinking becomes legal in all 50 states in the US. In general, we find that while alcohol use increases at age 21, there is limited evidence that marijuana use changes at age 21. The general direction of the effect was for a decrease in marijuana use among those that are marijuana-only users and an increase in marijuana use for those who are combination alcohol and marijuana users. Thus, one interpretation is that the data suggest both substitution and complementary effects. That is, among young adults who tend to use one substance, marijuana use decreases when alcohol use becomes more legally available. Among polysubstance using young adults, however, marijuana use increases when alcohol becomes legally available. This interpretation is made tenuous, however, by the lack of a significant change in slope based on the Joinpoint analysis. Thus, any effect observed is a small effect and caution is warranted in drawing conclusions about any substitution or complementary effect for marijuana when alcohol becomes legally available, at least among the high risk group assessed in the present study, drivers who died in motor vehicle crashes.

Most conservatively, we can at least conclude that once young adults reach the age when they can legally purchase and consume alcohol, the prevalence of alcohol use increases whereas the prevalence of marijuana use does not and trends in a negative direction for marijuana-only use. This finding is consistent with other evidence from national samples using the RD design (Crost and Guerrero, 2012; Yoruk and Yoruk, 2011), as well as other studies (Cameron and Williams, 2001; Chaloupka and Laixuthai, 1997; DiNardo and Lemieux, 2001; Thies and Register, 1993), documenting a substitution effect for alcohol and marijuana. We extend previous findings in several notable ways. Our data on motor vehicle crash decedents is notable, as it is a census of all deaths in the US with confirmed toxicology underlying drug measurement, and a population with high rates of substance use. Taken together, we would conservatively predict that increased availability of marijuana to young adults in US states that have passed medical and recreational use allowance may have positive spillover effects on alcohol, reducing use to some degree among young adults.

We note that our data are not consistent with a number of other studies (Chaloupka and Laixuthai, 1997; Farrelly et al. 2001; Pacula, 1998; Saffer and Chaloupka, 1999; Williams et al. 2004), which have found evidence for complementary effects of policies and laws on alcohol and marijuana use. Substantial epidemiological evidence indicates that individuals who use marijuana are more likely to drink alcohol compared with those who do not (Compton et al. 2005; Kessler et al. 2005), especially during young adulthood (McGue et al. 2001; Swendsen et al., 2012). The underlying causal mechanism for co-occurrence remains inadequately understood; co-occurrence may reflect shared genetic and environmental risk factors (Eaton et al. 2012; Krueger et al. 2007; Walton et al. 2011), and/or a causal sequence whereby some substances of abuse (e.g., alcohol) serve as ‘gateways’ to other substances of abuse (e.g., marijuana) (Huang et al. 2013; Kandel et al. 1992; Kandel et al. 2006; Levine et al. 2011). Regardless of the mechanism, co-occurrence across individuals supports the theory that substances are generally complementary. The use of the RD design, however, mitigates concerns about unmeasured non-comparability between drug users and non-users and in those exposed and unexposed to policies and law across states and time periods. We note, however, that there may be heterogeneity in the effects of laws and policies on alcohol and marijuana use; that is, for some laws and across some subgroups, alcohol and marijuana may be complementary. Meta-analysis and examination of effect heterogeneity would be helpful for future studies to fully understand potential public health implications of policy and law changes around substance use.

Study limitations are noted. The validity of the regression discontinuity design rests on the MLDA being the only exogenous source of variation in the change in alcohol use at age 21. That is, discontinuity occurring at the MLDA may not be attributable to MLDA if there are other sources of variation when individuals turn 21 that would create discontinuities. Further, the validity of the regression discontinuity design in the present study relies on assessment of one or more linear slopes. One could also model the relation between age and alcohol/marijuana use using non-linear models, complicating testing for discontinuity in the regression of age on these outcomes. Nonetheless, the interpretation of the relation between age and alcohol/marijuana use may not be completely attributable to MLDA. For example, if individuals are leaving secondary education institutions around this age, it could be that new social situations and/or the transition to full-time work or family life drive changes in alcohol and marijuana use, rather than the MLDA. In fact, we see a decrease in the slope of the relation between age and alcohol use after age 21, which is unlikely to be attributable to MLDA and rather to developmental changes co-occurring during this period. However, given that MLDA has well-documented and moderately large effects on alcohol consumption among young people, and the sharp discontinuity that is observed at age 21, it is unlikely that other role transitions drive these results. We note that states vary considerably in per capita consumption of alcohol (LaVallee and Yi, 2011) as we as availability, legality, and use of marijuana (Cerda et al. 2012; Wall et al. 2011). However, this state-level heterogeneity is unlikely to affect our results because the exposure of interest, MLDA, does not differ across states and we controlled for state where the crash occurred. Further, we had missing data on some covariates, including vehicle occupancy and race. Results controlling for these factors reduced the precision of our estimates, though the magnitudes of associations remain unchanged. Unless missing data is associated with alcohol and marijuana involved in the crash as well as age, we would expect increased precision but no change in the magnitude of our estimates if access to full data were available. Finally, we included states in which the proportion of decedents who were drug and alcohol tested was high and stable across all years of the study; we do not have information on whether drug and alcohol testing procedures differed across time. However, since we are averaging the effects of time across age, differences in procedures would not affect results unless the procedures were changed only for individuals in a certain age group.

## Conclusions

In conclusion, given the rapid changes currently underway in marijuana availability and price in the US, understanding the potential effects of increased use on other substances, as well as substance-related outcomes such as motor vehicle crash fatality, has never been more important. The weight of available evidence indicates that increasing access to marijuana may reduce alcohol use at the population level, though our results suggest that any negative trend in marijuana use is likely small. However, it should be noted that marijuana use is also a risk factor for involvement in fatal and nonfatal motor vehicle crashes (Li et al. 2013; Li et al. 2012; Romano et al. 2014); thus, the effects of increased marijuana use at the population level, while potential reducing alcohol use, may be null or even detrimental for fatality rates overall. Current data indicate that use of an illicit drug such as marijuana is less of a risk for fatality compared with the use of alcohol (Romano et al. 2014), though there is potential for changes in relative risks across drug type if marijuana use becomes increasingly common. Public health efforts to continue surveillance of drugged and drunk driving are critical at this important juncture in substance use policy in the US.

## Consent

This study was based on publicly available data about fatally injured drivers. The research protocol was reviewed by the institutional review board of the Columbia University Medical Center and was granted an exempt under 45 CFR 46 (not human subjects research).
